# Supplementary figures and images for: Mitochondria-related genes as prognostic signature of endometrial cancer and the effect of MACC1 on tumor cells
Source: PLoS One. 2025 May 12;20(5):e0323002. doi: 10.1371/journal.pone.0323002 (PMC12068703; doi:10.1371/journal.pone.0323002)

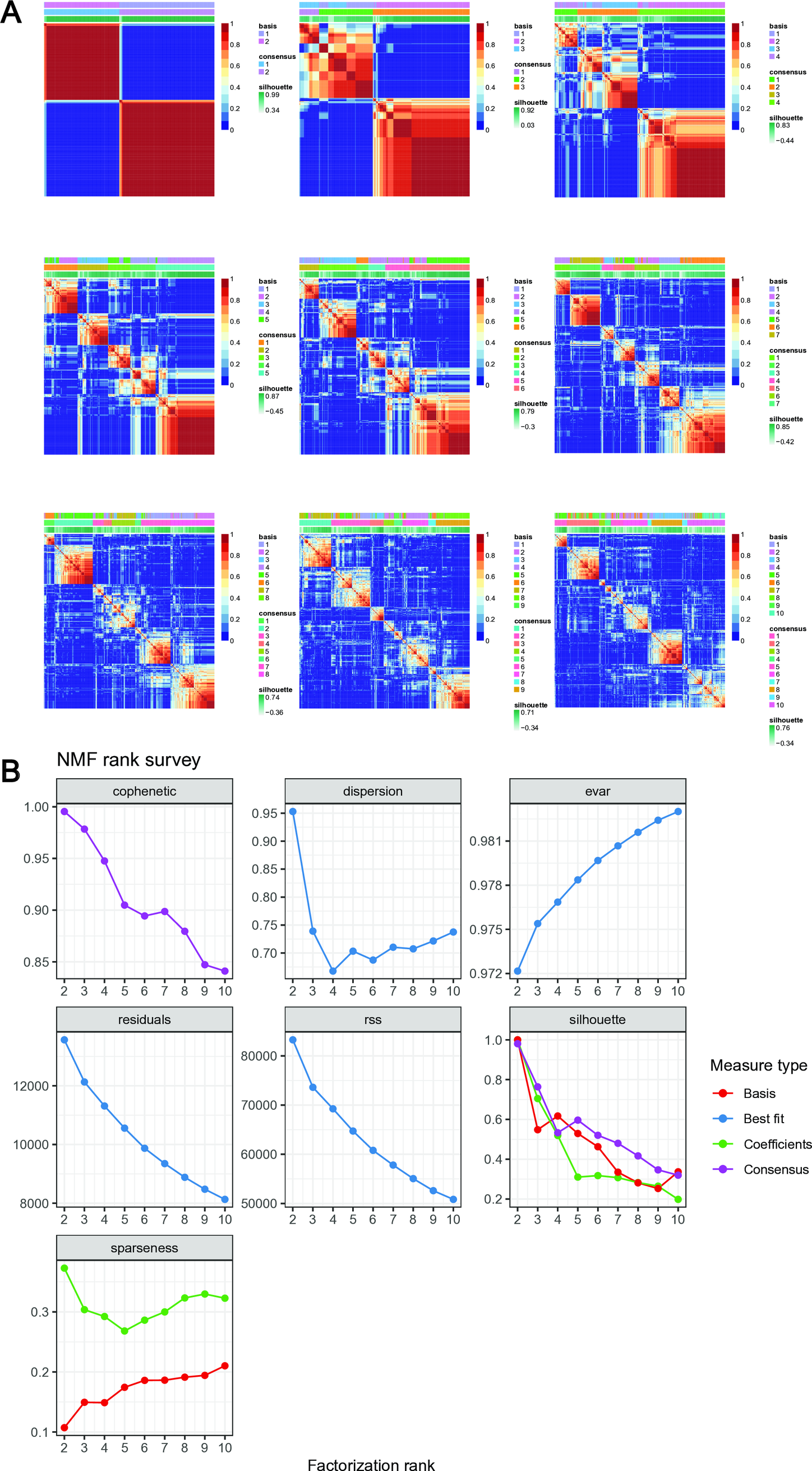

Supplement: S1 Fig — (TIFF) [file pone.0323002.s001.tif]

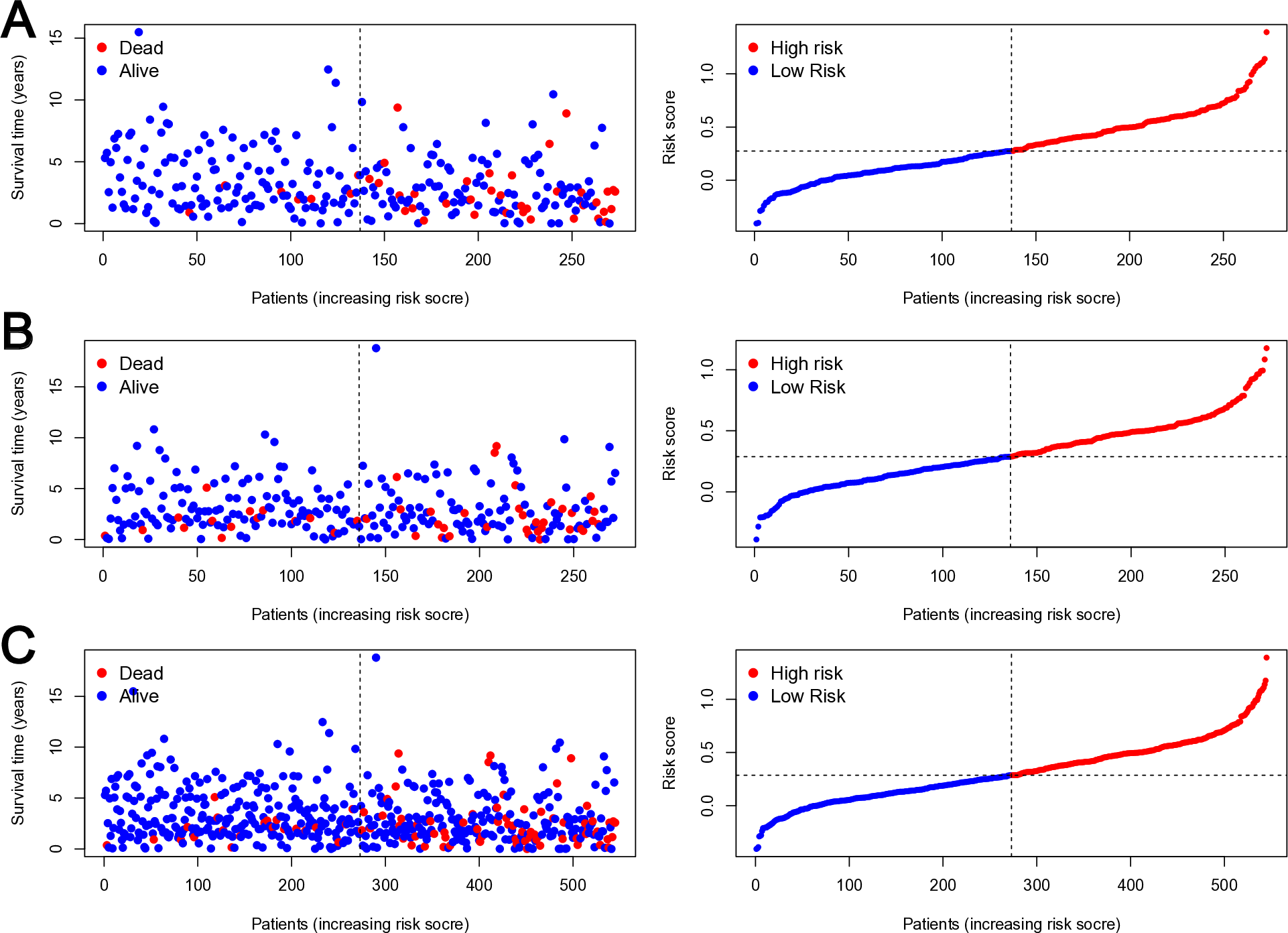

Supplement: S2 Fig — (TIFF) [file pone.0323002.s002.tif]

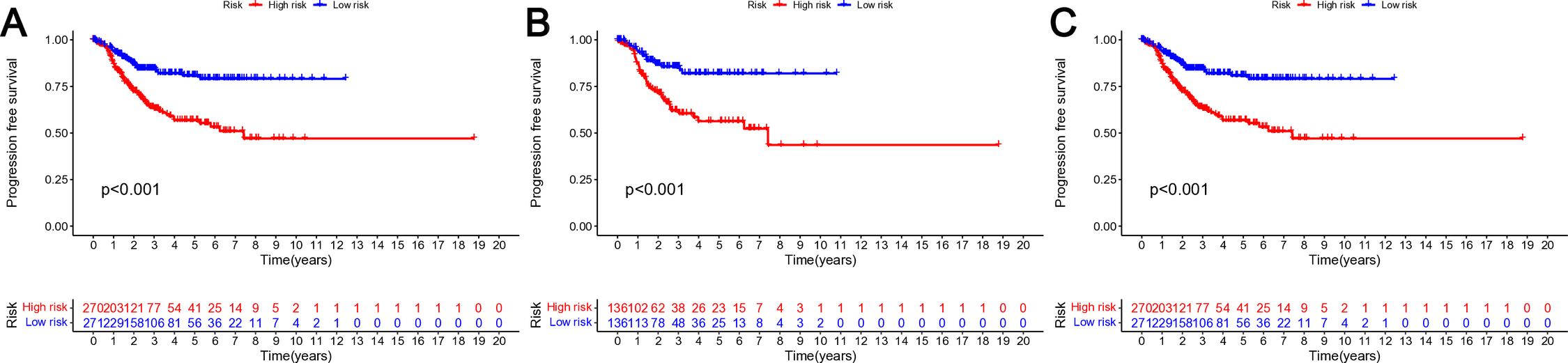

Supplement: S3 Fig — (TIFF) [file pone.0323002.s003.tif]

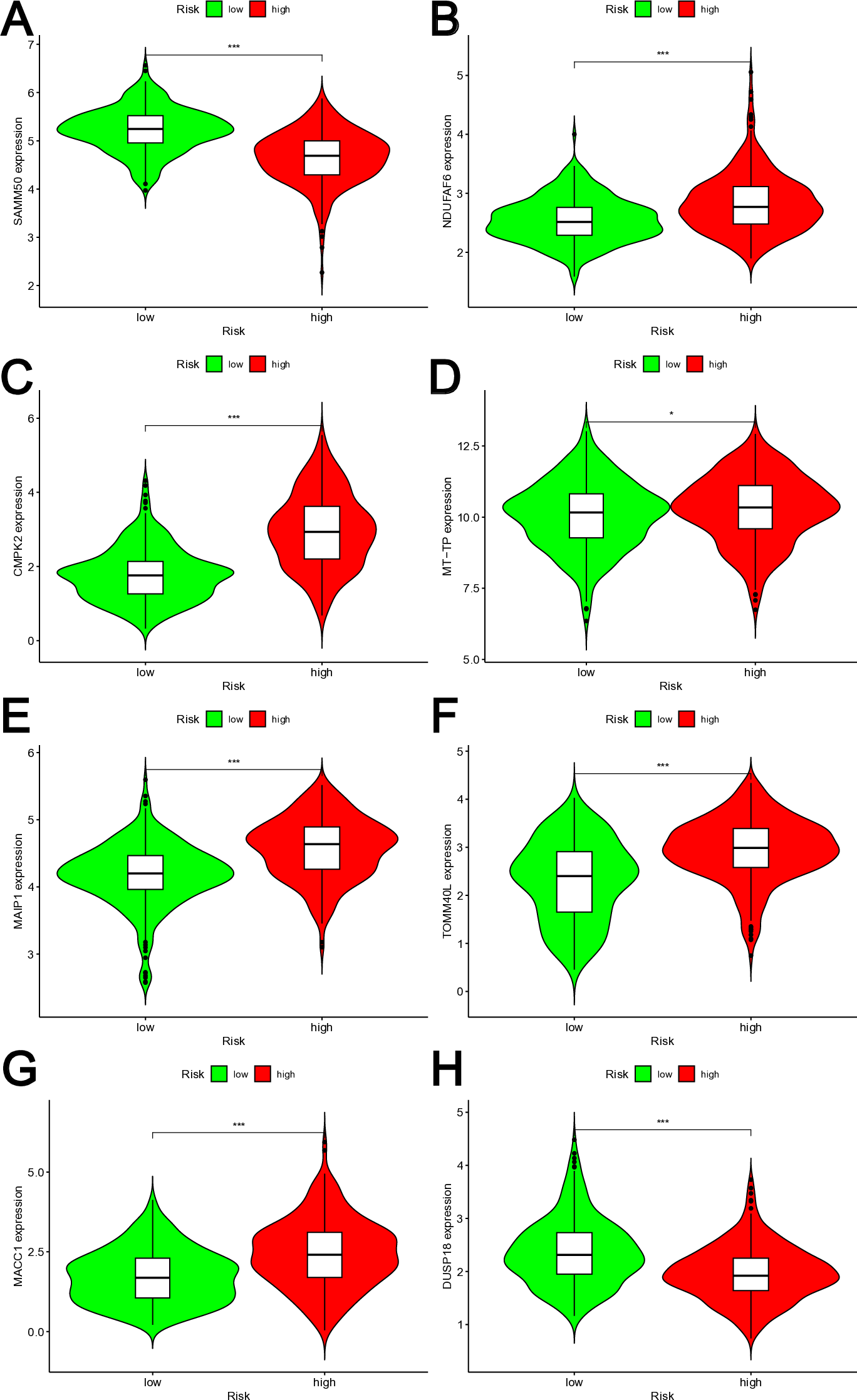

Supplement: S4 Fig — (A) SAMM50, (B) NDUFAF6, (C) CMPK2, (D) MT-TP, (E) MAIP1, (F) TOMM40L, (G) MACC1, (H) DUSP18. (TIFF) [file pone.0323002.s004.tif]

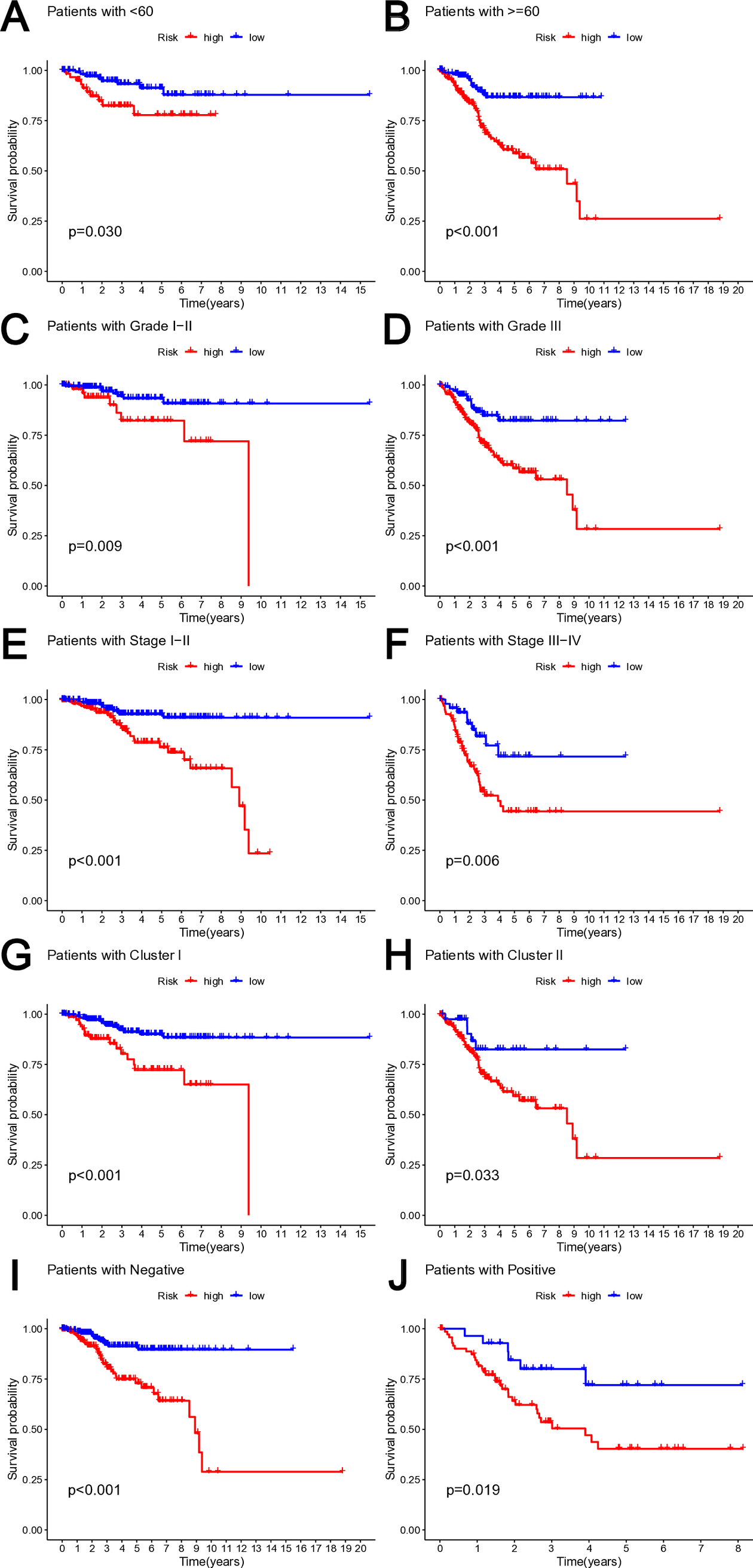

Supplement: S5 Fig — (A-H) The K-M survival curves of EC patients in the risk groups considering clinicopathology subgroups, including Age < 60 (A), Age>=60 (B), Grade I-II (C), Grade III (D), Stage I-II (E), Stage III-IV (F), Cluster I (G), and Cluster II (H), LNM Negative (I), and LNM Positive (J). (TIFF) [file pone.0323002.s005.tif]

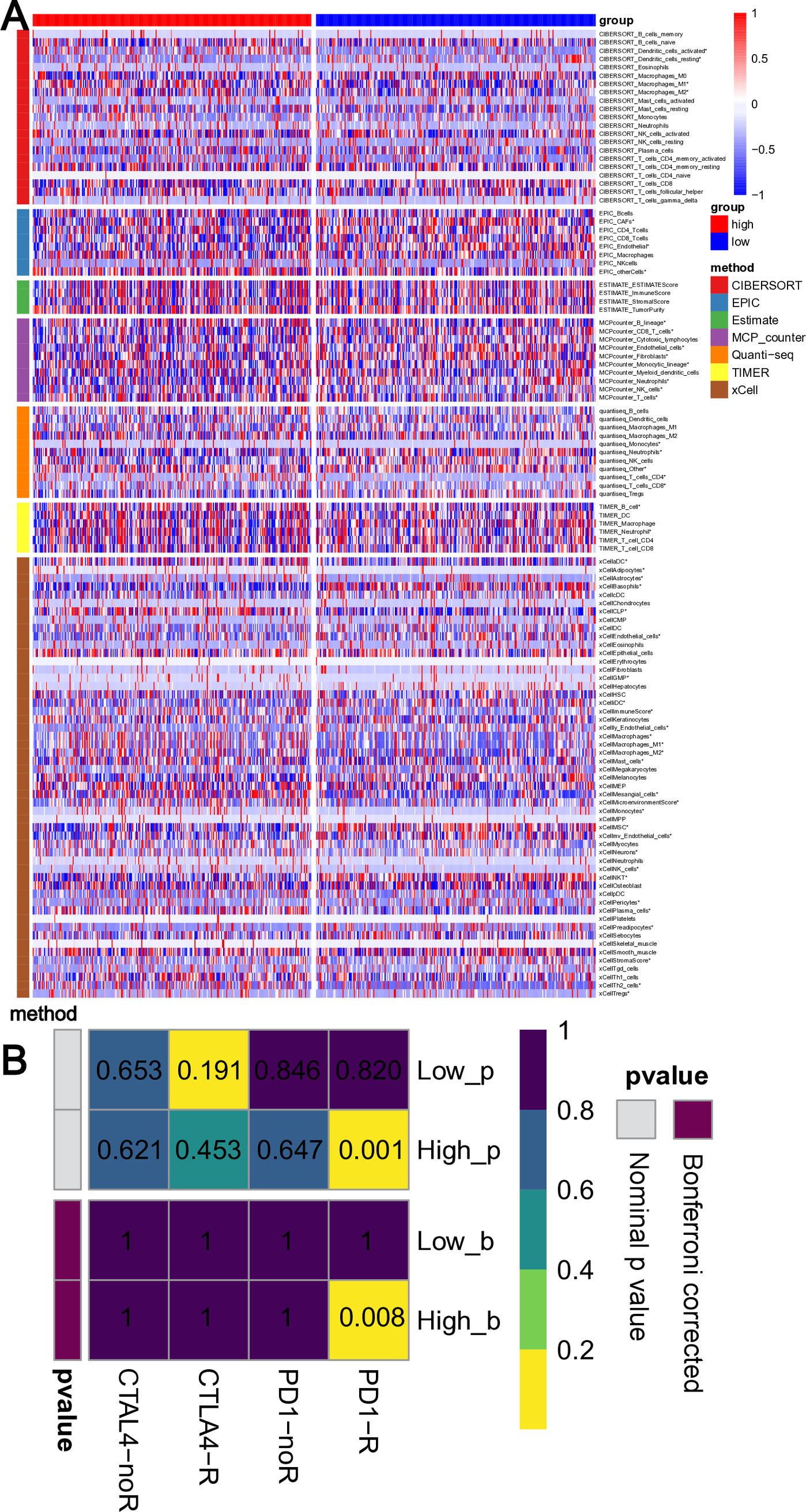

Supplement: S6 Fig — (A) Analysis of immune activity between the two risk groups using CIBERSORT, EPIC, ESITMATE, MCPcounter, quanTIseq, TIMER and xCell. *P < 0.05. (B) The subclass map showing the immunotherapeutic responses in different risk groups. (TIFF) [file pone.0323002.s006.tif]

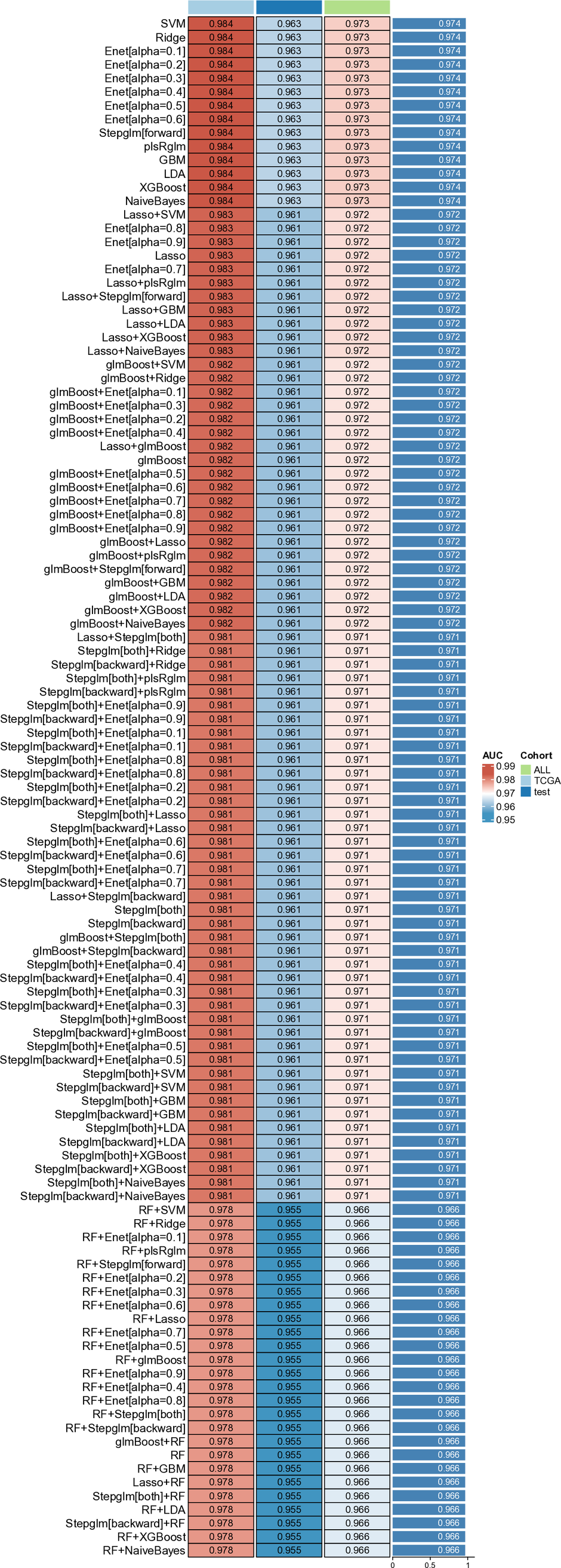

Supplement: S7 Fig — (TIFF) [file pone.0323002.s007.tif]

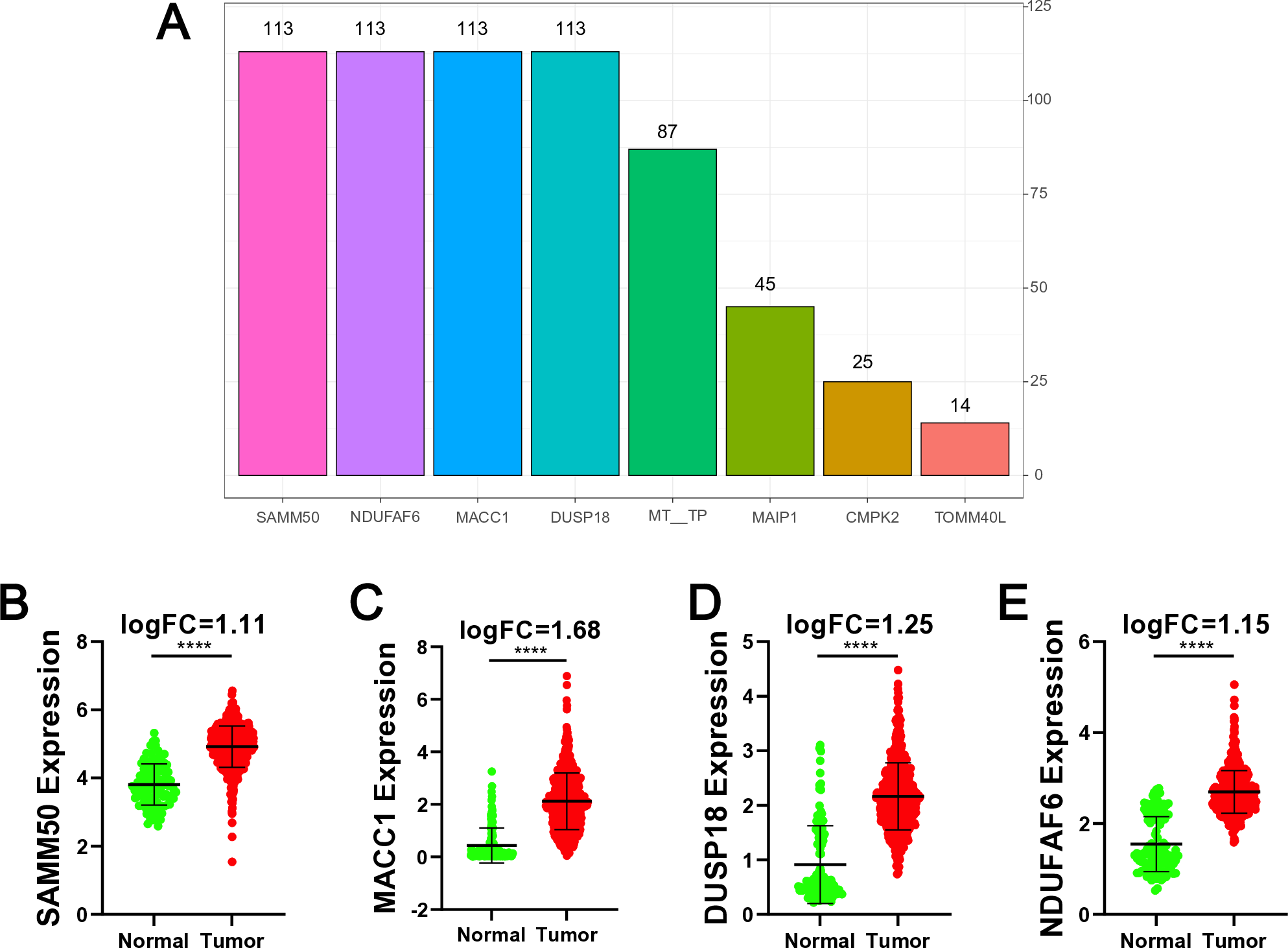

Supplement: S8 Fig — The expressed of SAMM50(B), MACC1(C), DUSP18(D) and NDUFAF6(E) in EC. (TIFF) [file pone.0323002.s008.tif]

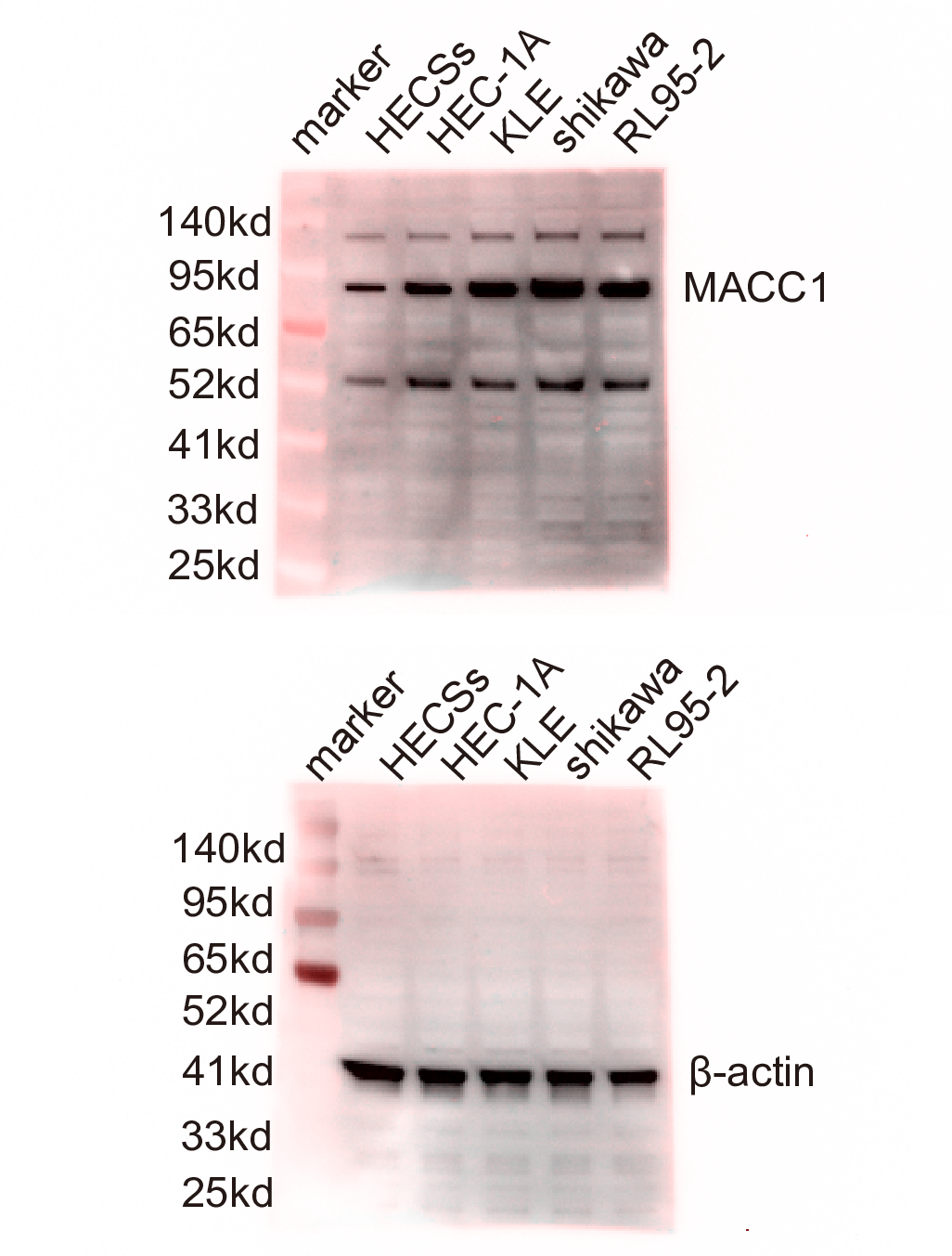

Supplement: S9 Fig — (TIFF) [file pone.0323002.s009.tif]

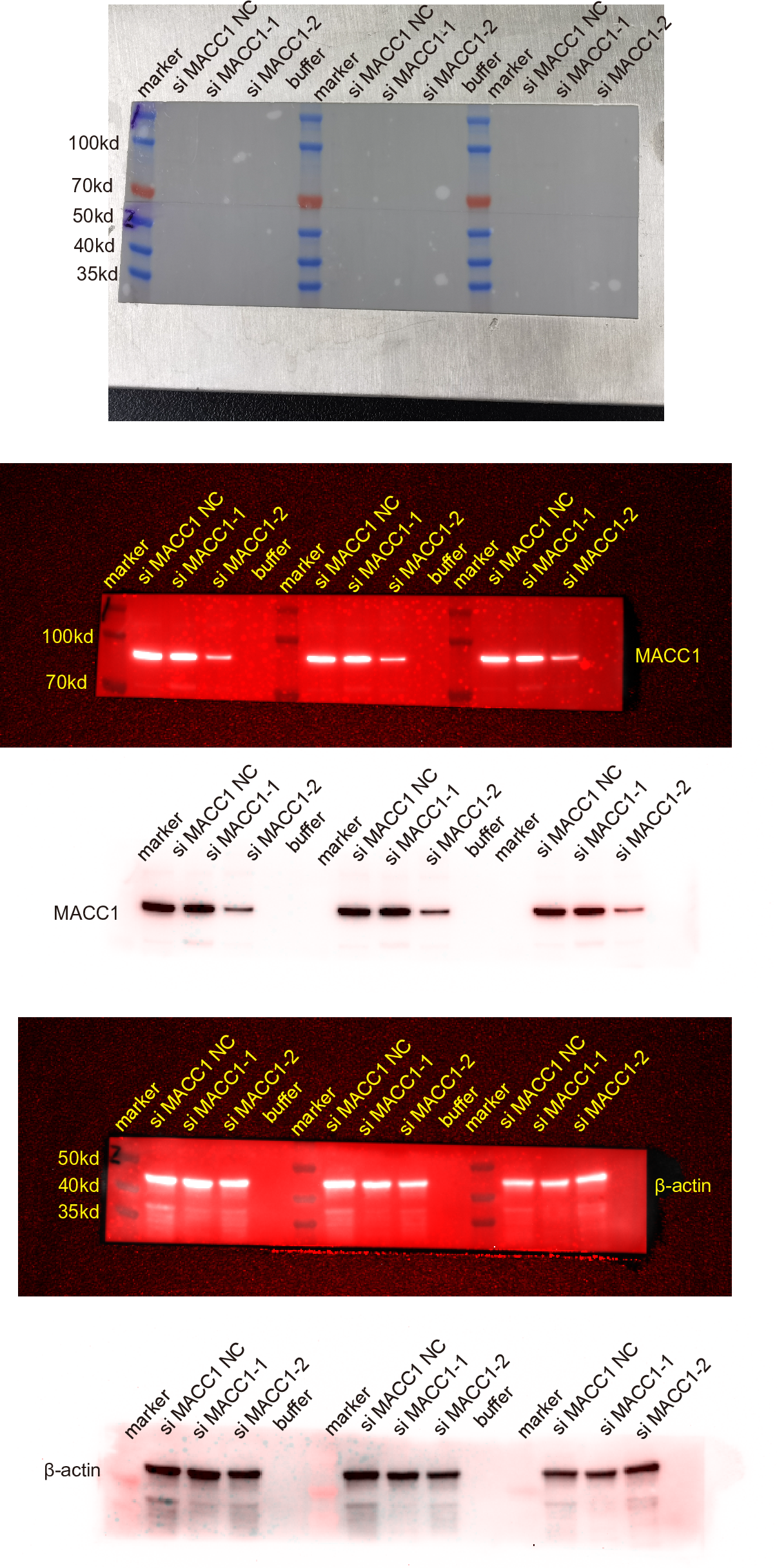

Supplement: S10 Fig — (TIFF) [file pone.0323002.s010.tif]

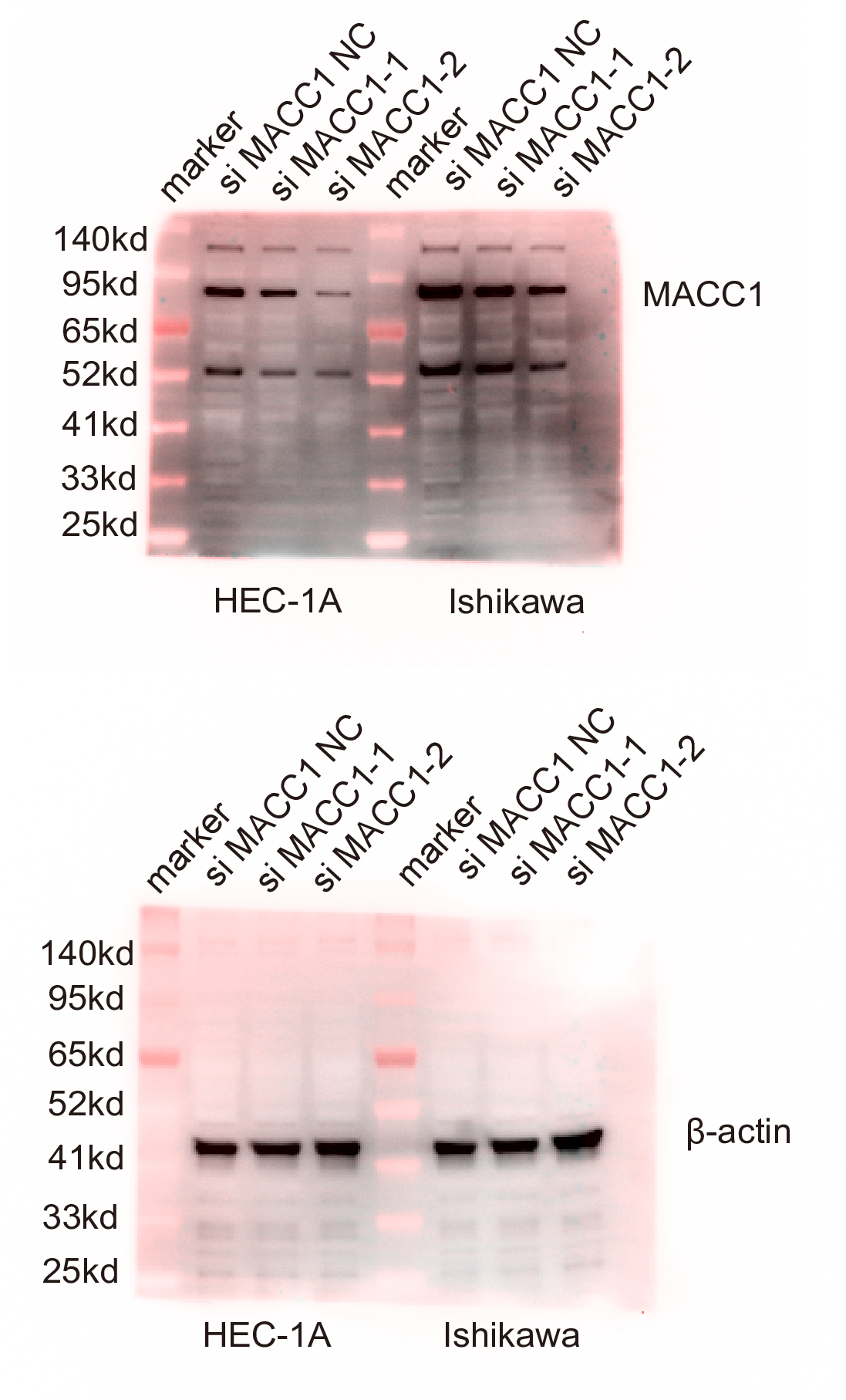

Supplement: S11 Fig — (TIFF) [file pone.0323002.s011.tif]
