# Supplementary material for: Mitochondria-related genes as prognostic signature of endometrial cancer and the effect of MACC1 on tumor cells
Source: PLoS One. 2025 May 12;20(5):e0323002. doi: 10.1371/journal.pone.0323002 (PMC12068703; doi:10.1371/journal.pone.0323002)
Supplement: Attachment — Submitted filename: Lin et al_PlosOne_reviewer assessment.pdf [file pone.0323002.s017.pdf]

The authors generated a prognostic signature for endometrial cancer patients and chose MACC1 as a key gene to further study in EC tumorigenesis. The study contains bioinformatic analysis and a figure on *in vitro* validation, and it would need to be further improved for its publication. There are some concerns that the authors should address:

Major concerns:

1) *In abstract: "Key MRGs with important features were identified using 113 algorithms and validated through colony formation, EdU proliferation, wound healing, apoptosis, transwell assays, and* ***animal studies****".*
   This line does not correlate with the figures presented here. There is only *in vitro* validation data for one MRG, MACC1, and there are no animal studies. In addition, there is a section for *in vivo* assays in materials and methods, and it is mentioned in several places. Still, in the last paragraph of the discussion the authors write "However, further *in vivo* experimental validation is necessary as this study has only been tested experimentally *in vitro*". The authors need to explain and either present the *in vivo* data or adapt the text accordingly.

2) The authors need to specify number of patients and characteristics of the cohort they used, as well as number of patients for each group of the KM curves. In addition, they need to validate their results in another cohort (for example, others available at the R2 database).

3) Lines 370-375. Please rephrase, it is not clear what the authors want to communicate here nor which the results they obtained are.

4) In general, the study needs to be explained better and the results more thoroughly described. It would also benefit from simplifying the figures, moving some to supplementary information, and homogenizing sizes and font sizes, and including a graphical abstract as summary.

   For instance, Figure 8A is hard to understand and follow, and in the text it is not described what the results actually are, only that "there are differences" (lines 413-417). The authors should describe these results and try to communicate the main points to the reader.

5) *Lines 432-436. The authors describe "According to our findings, a positive correlation was observed between the levels of MACC1 and CMPK2 and several drugs, including vinorelbine, cytarabine, docetaxel, tamoxifen, sorafenib, and crizotinib (Figure 9I). Conversely, an inverse relationship was identified between the levels of SAMM50 and NDUFAF6 and certain medications such as Paclitaxel and Sorafenib (Figure 9I)".*
   The correlation is not between genes and drugs, but with **drug susceptibility**. Please correct the language.

6) Lines 438-468. The title does not fit with the results since the panel "N" in Figure 10 does not exist. The authors should explain if this is a mistake or if they decided to remove the *in vivo* data and forgot to adapt the text.

7) Fig. 10F-G. The authors need to include additional EC cell lines, to have a panel that clearly shows that MACC1 is elevated in this type of cancer.

   Fig. 10 H-M. It would be convenient to show these experiments for si1, even if the knockdown efficiency is not as good as for si2, but it will prove the correlation between the levels of MACC1 and the effects on tumorigenesis.

   Figure 10I. This assay was performed at 14 days. Usually, the siRNAs last for a week or less. The authors should show a time-curse WB demonstrating that the knockdown of MACC1 is maintained throughout the experiment.

8) In the discussion, please include information on the current literature regarding metabolism in endometrial cancer, and the importance of your study in relation to it. In addition, please include a discussion on the possible therapeutic applicability of your study, and how it would be possible to target MACC1 (for instance, with MEK inhibitors).

Minor concerns:

- Line 90: Spell out "EEC".
- Line 186: Correct to "Cell culture"
- Figure 10J: Correct to "Hoechst" and include scale bar.
