# Supplementary material for: Mitochondria-related genes as prognostic signature of endometrial cancer and the effect of MACC1 on tumor cells
Source: PLoS One. 2025 May 12;20(5):e0323002. doi: 10.1371/journal.pone.0323002 (PMC12068703; doi:10.1371/journal.pone.0323002)
Supplement: Attachment — Submitted filename: Response to Reviewers.docx [file pone.0323002.s019.docx]

We gratefully appreciate the editors and all the reviewers for their time spend making their constructive remarks to our manuscript entitled “Mitochondria-related genes as prognostic signature of endometrial cancer and the effect of MACC1 on tumor cells”. Those comments are all valuable and very helpful for revising and improving our manuscript, as well as the important guiding significance to our study. The comments have been carefully studied and we have made necessary corrections in the hope of gaining approval. We have submitted our manuscript with tracked changes to highlight the revisions.

The Point-by-point response to reviewers are as follows:

**Reviewer 1**

**Comment**

The authors generated a prognostic signature for endometrial cancer patients and chose MACC1 as a key gene to further study in EC tumorigenesis. The study contains bioinformatic analysis and a figure on in vitro validation, and it would need to be further improved for its publication. There are some concerns that the authors should address:

Major concerns:

1) In abstract: “Key MRGs with important features were identified using 113 algorithms and validated through colony formation, EdU proliferation, wound healing, apoptosis, transwell assays, and animal studies”.

This line does not correlate with the figures presented here. There is only in vitro validation data for one MRG, MACC1, and there are no animal studies. In addition, there is a section for in vivo assays in materials and methods, and it is mentioned in several places. Still, in the last paragraph of the discussion the authors write “However, further in vivo experimental validation is necessary as this study has only been tested experimentally in vitro”. The authors need to explain and either present the in vivo data or adapt the text accordingly.

2) The authors need to specify number of patients and characteristics of the cohort they used, as well as number of patients for each group of the KM curves. In addition, they need to validate their results in another cohort (for example, others available at the R2 database).

3) Lines 370-375. Please rephrase, it is not clear what the authors want to communicate here nor which the results they obtained are.

4) In general, the study needs to be explained better and the results more thoroughly described. It would also benefit from simplifying the figures, moving some to supplementary information, and homogenizing sizes and font sizes, and including a graphical abstract as summary.

For instance, Figure 8A is hard to understand and follow, and in the text it is not described what the results actually are, only that “there are differences” (lines 413-417). The authors should describe these results and try to communicate the main points to the reader.

5) Lines 432-436. The authors describe “According to our findings, a positive correlation was observed between the levels of MACC1 and CMPK2 and several drugs, including vinorelbine, cytarabine, docetaxel, tamoxifen, sorafenib, and crizotinib (Figure 9I). Conversely, an inverse relationship was identified between the levels of SAMM50 and NDUFAF6 and certain medications such as Paclitaxel and Sorafenib (Figure 9I)”.

The correlation is not between genes and drugs, but with drug susceptibility. Please correct the language.

6) Lines 438-468. The title does not fit with the results since the panel “N” in Figure 10 does not exist. The authors should explain if this is a mistake or if they decided to remove the in vivo data and forgot to adapt the text.

7) Fig. 10F-G. The authors need to include additional EC cell lines, to have a panel that clearly shows that MACC1 is elevated in this type of cancer.

Fig. 10 H-M. It would be convenient to show these experiments for si1, even if the knockdown efficiency is not as good as for si2, but it will prove the correlation between the levels of MACC1 and the effects on tumorigenesis.

Figure 10I. This assay was performed at 14 days. Usually, the siRNAs last for a week or less. The authors should show a time-curse WB demonstrating that the knockdown of MACC1 is maintained throughout the experiment.

8) In the discussion, please include information on the current literature regarding metabolism in endometrial cancer, and the importance of your study in relation to it. In addition, please include a discussion on the possible therapeutic applicability of your study, and how it would be possible to target MACC1 (for instance, with MEK inhibitors).

Minor concerns:

Line 90: Spell out “EEC”.

Line 186: Correct to “Cell culture”

Figure 10J: Correct to “Hoechst” and include scale bar.

**Response**

Dear Reviewer,

We gratefully appreciate you for your time spend making your constructive remarks to our manuscript. Your comments are all valuable and very helpful for revising and improving our manuscript, as well as the important guiding significance to our study. The comments have been carefully studied and we have made necessary corrections in the hope of gaining approval. We have submitted our manuscript with tracked changes to highlight the revisions.

**Comment 1**

In abstract: “Key MRGs with important features were identified using 113 algorithms and validated through colony formation, EdU proliferation, wound healing, apoptosis, transwell assays, and animal studies”.

This line does not correlate with the figures presented here. There is only in vitro validation data for one MRG, MACC1, and there are no animal studies. In addition, there is a section for in vivo assays in materials and methods, and it is mentioned in several places. Still, in the last paragraph of the discussion the authors write “However, further in vivo experimental validation is necessary as this study has only been tested experimentally in vitro”. The authors need to explain and either present the in vivo data or adapt the text accordingly.

**Response 1**

We sincerely appreciate your constructive comments. We would like to address these concerns as follows:

1. Regarding the MRG validation:

- We have revised the phrasing in the abstract to clarify that MACC1 was selected as the key MRG for experimental validation based on our comprehensive bioinformatic analysis.

- The rationale for selecting MACC1 is thoroughly explained in the Results section.

*Line 483-513*

2. Regarding the animal experimental data:

- Due to our oversight during submission, we inadvertently omitted the figure containing animal experimental data.

- We have now included these data as Fig 8 in the revised manuscript.

3. We have carefully reviewed and revised the entire manuscript to ensure:

- Consistent description of the experimental validation throughout the text.

- Accurate representation of both in vitro and in vivo findings.

- Removal of the incorrect statement in the discussion about "only in vitro validation".

*Line 40-43:*

Systematic experimental validation, including both in vitro and in vivo approaches, demonstrated that MACC1 downregulation significantly suppressed EC progression, highlighting its potential as a therapeutic target.

*Line 632-637:*

Additionally, although we conducted both in vivo and in vitro experiments, the precise molecular mechanism by which MACC1 promotes endometrial cancer progression remains to be fully elucidated. Further investigation is needed to comprehensively understand the role of MACC1 in EC pathogenesis, which could potentially improve diagnostic accuracy, treatment strategies, and prognostic evaluation.

**Comment 2**

2) The authors need to specify number of patients and characteristics of the cohort they used, as well as number of patients for each group of the KM curves. In addition, they need to validate their results in another cohort (for example, others available at the R2 database).

**Response 2**

We appreciate your constructive comments. We acknowledge the oversight in our original manuscript regarding patient information and have made the following clarifications:

1. Patient Information and Cohort Characteristics:

- The total number of participants in our study was 545 primary EC patients and 113 healthy controls.

- The detailed patient characteristics (including age, FIGO stage, grade, etc.) have been added to S1 Table in the revised manuscript.

- For the KM curves analysis, patient numbers are clearly shown below each curve.

2. Regarding External Validation:

We made extensive efforts to validate our findings using external datasets:

a) Public Database Search:

- GEO database (https://www.ncbi.nlm.nih.gov/geo/)

- R2 Genomics Analysis Platform (https://hgserver1.amc.nl/cgi-bin/r2/main.cgi)

- TCGA and GTEx databases (which we have already included in our analysis)

b) Commercial Microarray Resources:

- Outdo Biotech Ltd (<https://outdoivd.com/biology/cdna.html>)

- DAIXUE Bio Ltd (<http://www.biotechdx.com/plus/list.php?tid=16>)

- Shanghai Biochip Ltd (<https://www.shbiochip.com/wzsy>)

- Beijing Bionacil Biotechnology Ltd (<http://bionaxin.com/sy>)

Unfortunately, none of these sources contained endometrial cancer datasets with the necessary prognostic information for model validation. This limitation is primarily due to the current scarcity of publicly available endometrial cancer datasets with complete survival data. We have acknowledged this limitation in the Discussion section of our manuscript.

**Comment 3**

3) Lines 370-375. Please rephrase, it is not clear what the authors want to communicate here nor which the results they obtained are.

**Response 3**

We appreciate your constructive comments. To address this concern, we have thoroughly revised this section to enhance clarity. The core objective of this analysis was to evaluate the prognostic value of our MRG-related signature across different clinicopathological subgroups. We have modified the text as follows:

*Line 378-385:*

To comprehensively validate the prognostic robustness of our MRG-related signature, we conducted stratified survival analyses across key clinicopathological features, including age (<60 vs. ≥60 years, S5 Fig A-B), tumor grade (G1-2 vs. G3, S5 Fig C-D), FIGO stage (I-II vs. III-IV, S5 Fig E-F), MRG-related clusters (S5 Fig G-H), and lymph node status (positive vs. negative, S5 Fig I-J). Notably, the signature maintained its prognostic significance within each subgroup, consistently demonstrating significantly poorer overall survival in high-risk patients compared to their low-risk counterparts (all P < 0.05).

**Comment 4**

4) In general, the study needs to be explained better and the results more thoroughly described. It would also benefit from simplifying the figures, moving some to supplementary information, and homogenizing sizes and font sizes, and including a graphical abstract as summary.

For instance, Figure 8A is hard to understand and follow, and in the text it is not described what the results actually are, only that “there are differences” (lines 413-417). The authors should describe these results and try to communicate the main points to the reader.

**Response 4**

We sincerely appreciate your constructive suggestions for improving the manuscript's clarity and presentation. We have implemented comprehensive changes as follows:

1. Figure Organization and Presentation:

- Reorganized figures by moving detailed technical analyses to supplementary materials.

- Standardized all figures following PLOS ONE guidelines, including consistent font sizes and formatting.

- Created a graphical abstract to provide a clear visual summary of our study design and key findings.

2. Enhanced Results Description:

*Lines 430-453* have been revised to provide a more detailed explanation:

"To systematically characterize the immune landscape associated with our risk signature, we employed seven complementary computational deconvolution methods (CIBERSORT, EPIC, ESTIMATE, MCPcounter, quanTIseq, TIMER, and xCell) to profile the tumor immune microenvironment (S6 Fig A). This multi-algorithmic approach enabled robust and comprehensive evaluation of immune cell infiltration patterns. Our analysis revealed striking differences in immune cell composition between high- and low-risk groups. Notably, we observed significant enrichment of multiple immune cell populations in the high-risk group, particularly in adaptive immune components. Specifically, both CD4+ and CD8+ T cell populations showed consistent elevation across multiple algorithms (MCPcounter_T_cells, MCPcounter_CD8_T_cells), suggesting enhanced T cell infiltration in high-risk tumors. Intriguingly, we observed substantial differences in stromal components, particularly in endothelial cells and cancer-associated fibroblasts (EPIC_CAFs, EPIC_Endothelial, MCPcounter_Endothelial_cells, xCellmv_Endothelial_cells). This finding suggests that the risk signature not only reflects immune cell dynamics but also encompasses broader changes in the tumor microenvironment. The ESTIMATE scores further corroborated these findings, indicating significant differences in both immune and stromal components between risk groups. Of particular interest was the enhanced NK cell signature (MCPcounter_NK_cells) in the high-risk group, suggesting increased natural cytotoxicity potential. This observation, combined with the elevated T cell signatures, indicates a more active but potentially dysregulated immune response in high-risk tumors. Notably, these findings suggest that high-risk patients might be particularly responsive to immunotherapy approaches, specifically PD1-R therapy, as supported by our additional analysis (S6 Fig B)."

3. Figure Simplification:

- Former Figure 8A (now S6 Fig A) has been redesigned for better clarity

- Added clear annotations and legends

- Included statistical significance indicators

- Provided a detailed figure legend explaining the analysis methods and key findings.

These revisions aim to enhance the manuscript's readability while ensuring that our scientific findings are presented in a clear and accessible manner.

**Comment 5**

5) Lines 432-436. The authors describe “According to our findings, a positive correlation was observed between the levels of MACC1 and CMPK2 and several drugs, including vinorelbine, cytarabine, docetaxel, tamoxifen, sorafenib, and crizotinib (Figure 9I). Conversely, an inverse relationship was identified between the levels of SAMM50 and NDUFAF6 and certain medications such as Paclitaxel and Sorafenib (Figure 9I)”.

The correlation is not between genes and drugs, but with drug susceptibility. Please correct the language.

**Response 5**

Thank you for this important observation regarding the precise terminology in describing drug-gene relationships. We fully agree that accuracy in describing pharmacogenomic relationships is crucial, particularly in the context of drug susceptibility and treatment response. We have revised the text to more accurately reflect the relationship between gene expression levels and drug susceptibility:

*Line 465-474:*

According to our findings, the genes in the model were associated with drug susceptibility. A positive correlation was observed between the levels of MACC1 and CMPK2 and the drug resistance scores calculated by oncoPredict for several medications, including vinorelbine, cytarabine, docetaxel, tamoxifen, sorafenib, and crizotinib (Figure 6I). This indicates that higher expression levels of MACC1 and CMPK2 may be associated with decreased drug sensitivity to these drugs. Conversely, an inverse relationship was identified between the levels of SAMM50 and NDUFAF6 and the drug resistance scores for certain medications, such as Paclitaxel and Sorafenib (Figure 6I). This suggests that higher expression levels of SAMM50 and NDUFAF6 may be linked to increased drug sensitivity to these drugs.

**Comment 6**

6) Lines 438-468. The title does not fit with the results since the panel “N” in Figure 10 does not exist. The authors should explain if this is a mistake or if they decided to remove the in vivo data and forgot to adapt the text.

**Response 6**

Thank you for your careful observation regarding this discrepancy. We sincerely apologize for this oversight in our submission. You are correct that there was a mismatch between the manuscript text and Figure 10, where panel "N" was referenced but not included. This occurred during the file preparation process where we inadvertently uploaded an incomplete version of Figure 10 that did not contain the in vivo experimental data. We have now rectified this error by:

1. Uploading the complete version of Figure 8 (the original Figure 10) that includes all panels, including the in vivo experimental data (panel N)

2. Ensuring consistency between the figure and the corresponding text description in *Lines 509-513.*

This correction maintains the integrity of our original research findings while properly presenting all the experimental data referenced in the manuscript. We appreciate your attention to detail which helped us identify and correct this technical oversight in our submission.

**Comment 7**

7) Fig. 10F-G. The authors need to include additional EC cell lines, to have a panel that clearly shows that MACC1 is elevated in this type of cancer.

Fig. 10 H-M. It would be convenient to show these experiments for si1, even if the knockdown efficiency is not as good as for si2, but it will prove the correlation between the levels of MACC1 and the effects on tumorigenesis.

Figure 10I. This assay was performed at 14 days. Usually, the siRNAs last for a week or less. The authors should show a time-curse WB demonstrating that the knockdown of MACC1 is maintained throughout the experiment.

**Response 7**

Thank you for your constructive suggestions. We have addressed each of your concerns as follows:

1. Regarding MACC1 expression in EC cell lines:

We have expanded our analysis to include additional endometrial cancer cell lines. Specifically, we performed both RT-qPCR and Western blot analyses on four EC cell lines (HEC-1A, Ishikawa, RL95-2, and KLE) and normal endometrial cells (HESCs) as controls (Figures 7A-B). The results consistently demonstrated elevated MACC1 expression across EC cell lines compared to HESCs, strengthening our findings on MACC1's role in endometrial cancer.

2. Regarding siRNA-1 experiments:

Following your suggestion, we have now included parallel experiments using both siRNA-1 and siRNA-2. Despite siRNA-1's lower knockdown efficiency, we observed consistent phenotypic effects in Cell culture experiments. These additional data further validate the correlation between MACC1 expression levels and tumorigenesis.

3. Regarding the duration of siRNA knockdown:

To address the concern about siRNA stability in the 14-day colony formation assay, we have validated the knockdown effect at both protein and mRNA levels at day14 post-transfection (Figure 7G). Our data show that under our optimized experimental conditions, we achieved sustained MACC1 knockdown throughout the 14-day period. This is consistent with several published studies that have successfully used siRNA approaches in similar long-term assays (PMID: 35524319, 38038147, 33061365, 33000203, 37656231).

*Line 157-162:*

Meisen Chinese Tissue Culture Collections (Zhejiang, China) provided the commonly utilised EC cell lines HEC-1A, Ishikawa, RL95-2 and KLE, as well as human endometrial stromal cells (HESCs). Ishikawa cells were cultured in 90% RPMI 1640 medium plus 10% FBS, while HEC-1A cells were grown in 90% McCoy's 5A media plus 10% FBS (PAN-Biotech, Germany). RL95-2 and KLE cells were cultured in 90% DMEM medium plus 10% FBS. Cells were cultured at 37°C in a 5% CO_2_ atmosphere.

*Line 496-499:*

To investigate the effect of MACC1 on tumor, we transfected EC cells with MACC1-siRNA. MACC1-siRNA-2 (si2) showed better efficacy than MACC1-siRNA-1(si1) in the evaluation of knockdown efficiency.

**Comment 8**

8) In the discussion, please include information on the current literature regarding metabolism in endometrial cancer, and the importance of your study in relation to it. In addition, please include a discussion on the possible therapeutic applicability of your study, and how it would be possible to target MACC1 (for instance, with MEK inhibitors).

Minor concerns:

Line 90: Spell out “EEC”.

Line 186: Correct to “Cell culture”

Figure 10J: Correct to “Hoechst” and include scale bar.

**Response 8**

Thank you for your constructive comments. We have made a comprehensive revision to the discussion section to emphasize the relationship between endometrial cancer and metabolism, as well as the significance of this study. We have also added details on the potential therapeutic applicability of this research and how MACC1 may be targeted. Additionally, we have made the necessary corrections to address the errors you pointed out.

*Line 553-565:*

EC metabolism has emerged as a crucial area of research in recent years, with studies revealing altered metabolic pathways that contribute to its development and progression. For instance, metabolic reprogramming, such as increased aerobic glycolysis and fatty acid synthesis, has been implicated in EC. Eskander et al. discussed the integration of immunotherapy into first-line treatment for advanced and metastatic endometrial cancer, highlighting the evolving therapeutic strategies that potentially target metabolic pathways. The significance of research in this area cannot be overstated, given the rising incidence and mortality of endometrial cancer, coupled with the limited success of current treatment options. By investigating the specific mechanisms through which metabolic pathways contribute to tumorigenesis and progression, researchers can identify potential therapeutic targets and develop innovative treatment strategies. This could pave the way for more effective and personalized treatments for endometrial cancer, ultimately improving patient outcomes.

*Line 599-608:*

The involvement of MACC1 in the HGF/c-Met signaling pathway is a critical mechanism driving its oncogenic effects. MACC1 facilitates the epithelial-mesenchymal transition (EMT), cell invasion, and metastasis by regulating the HGF/c-Met signaling pathway. This pathway, which promotes tumor cell survival and migration, is frequently upregulated in EC and other malignancies. Targeting the HGF/c-Met axis, along with MACC1, offers a promising strategy for inhibiting tumor progression. Notably, MEK inhibitors, such as trametinib, have shown efficacy in suppressing the HGF/c-Met signaling pathway by blocking downstream ERK signaling, which is regulated by MACC1, this provides a rationale for combining MEK inhibitors with therapies targeting MACC1 to potentiate the therapeutic response in EC.

*Line 612-620:*

The role of MACC1 in cancer progression and metastasis makes it a promising therapeutic target. As highlighted in our study, MACC1 is significantly overexpressed in EC cells, and its elevated levels correlate with poorer prognosis, suggesting its potential as a biomarker for prognosis and a target for treatment. While MACC1 has been well studied in several cancers, including colon, gastric, and lung cancers, its role in EC remains less explored. Our findings demonstrate that MACC1 knockdown effectively suppresses tumor migration, invasion, and proliferation, highlighting its functional importance in EC progression.

*Line 621-627:*

The integration of metabolic reprogramming as a therapeutic approach in EC is also becoming increasingly important. As discussed, metabolic shifts in EC cells, such as increased aerobic glycolysis and fatty acid synthesis, contribute to tumor progression. Targeting these altered metabolic pathways in combination with MACC1 inhibition could further enhance treatment efficacy. In particular, the regulation of mitochondrial function and ROS production, both of which are influenced by MACC1, represents another potential avenue for therapeutic intervention.

**Reviewer 2**

**Comment**

In this manuscript, the authors create a prognostic model for endometrial cancer using mitochondria-related genes (MRGs) to predict patient outcomes, drug sensitivity, and immunotherapy response. They identify of the gene MACC1 as a marker for endometrial cancer progression, demonstrating that MACC1 downregulation reduces proliferation, migration and invasion, making it a potential therapeutic target. I have the following criticisms that the authors might address:

Major comments:

- Introduction, lines 115–117, the authors write: “however, it remains unclear how individuals with endometrial cancer would respond to genes associated with these organelles”. What do the authors mean by responding to genes? Respond to mutations or if these genes are targeted?

- It is unclear if in Figure 1A the MRGs in EC were compared to healthy tissue. Otherwise, how can you discern if a gene is upregulated or downregulated?

- It would be of great help for the reader if the authors spelled out the MRGs selected for the risk score. In addition, for MACC1, a description of its function would be beneficial also in the results part to understand the following experiments in vitro.

- In Figure 10B, the authors analyze in silico the expression of the different selected markers (SAMM50, NDUFAF6, MACC1, and DUSP18) in normal vs tumor tissues. In accordance with their previous results, these markers should be higher expressed in high-risk samples. Is the expression of these genes elevated in higher tumor stages compared to low risk classified tumors? This could be assessed by dividing the tumors in low and high risk and comparing gene expression.

- Figure 10N, regarding the impact on in vivo tumor growth of MACC1 downregulation, is not included in the submitted version.

- In the discussion, the manuscript would benefit from a correlation between the results observed in the in vitro experiments downregulating MACC1 and what it is known about its function regulating migration, epithelial to mesenchymal transition and cytoskeleton dynamics.

- Even though the authors describe in vivo work in the last part of the results, the methods section of that experiment is described in detailed, and mention Figure 10N which is not included, in the discussion it is written that this study has only been tested experimentally in vitro.

Minor comments:

- Introduction, line 99 – 100, the authors write: “Mitochondria take up substrates from the cytoplasm and use them for important life activities (6).” In this context, it would be better to change “life activities” for “cellular processes”

- Materials and methods, line 186: correct to cell culture.

**Response**

Dear Reviewer,

We gratefully appreciate you for your time spend making your constructive remarks to our manuscript. Your comments are all valuable and very helpful for revising and improving our manuscript, as well as the important guiding significance to our study. The comments have been carefully studied and we have made necessary corrections in the hope of gaining approval. We have submitted our manuscript with tracked changes to highlight the revisions.

**Comment 1**

Introduction, lines 115–117, the authors write: “however, it remains unclear how individuals with endometrial cancer would respond to genes associated with these organelles”. What do the authors mean by responding to genes? Respond to mutations or if these genes are targeted?

**Response 1**

Thank you for highlighting this imprecise wording. You are absolutely right that the original phrasing "respond to genes" was ambiguous and scientifically inaccurate. We have revised this sentence to more precisely convey our intended meaning:

*Line 81-82:*

However, the role of organelle-related genes in endometrial cancer is still unclear.

**Comment 2**

It is unclear if in Figure 1A the MRGs in EC were compared to healthy tissue. Otherwise, how can you discern if a gene is upregulated or downregulated?

**Response 2**

Thank you for this important question regarding the comparative analysis in Figure 1A. We apologize for not clearly describing the methodology of our differential expression analysis. To clarify:

The analysis in Figure 1A represents a comparative study between:

- Endometrial cancer tissues (n=545) from TCGA database

- healthy tissues from TCGA (n=35) and GTEx databases

We performed differential expression analysis of mitochondria-related genes (MRGs) between these two groups using the following criteria:|log2 fold change|>1, Adjusted p-value < 0.05.

This comparison between cancerous and normal tissues allowed us to accurately identify upregulated and downregulated MRGs in endometrial cancer. We have now updated the figure legend and methods section to explicitly state these sample numbers and analysis parameters for better clarity and reproducibility.

*Line 95-99:*

We excluded primary EC samples with inadequate clinical data or follow-up information, ultimately including 545 primary EC samples and 113 healthy controls in our study. From these data, we extracted patient characteristics including age, pathological grade, pathological stage, and lymph node metastasis status for further analysis.

*Line 108-109:*

We employed the limma package to perform differential expression analysis of MRGs in EC samples and normal tissues (P<0.05 and |logFC|>1).

**Comment 3**

It would be of great help for the reader if the authors spelled out the MRGs selected for the risk score. In addition, for MACC1, a description of its function would be beneficial also in the results part to understand the following experiments in vitro.

**Response 3**

We appreciated your constructive comments. We have uploaded the supplementary information for the MRGs used in the risk score. The list for this part can be found in **S3 Table**.

*Line 116-117:*

The MRGs used for risk score can be found in the supplementary information **S3 Table**.

**Comment 4**

In Figure 10B, the authors analyze in silico the expression of the different selected markers (SAMM50, NDUFAF6, MACC1, and DUSP18) in normal vs tumor tissues. In accordance with their previous results, these markers should be higher expressed in high-risk samples. Is the expression of these genes elevated in higher tumor stages compared to low risk classified tumors? This could be assessed by dividing the tumors in low and high risk and comparing gene expression.

**Response 4**

Thank you for this insightful question regarding the relationship between gene expression and risk classification. We would like to clarify several important points:

1. Expression Pattern in Tumor vs. Normal Tissues:

Based on our TCGA database analysis, we confirmed that SAMM50, NDUFAF6, MACC1, and DUSP18 show differential expression between tumor and normal tissues. However, we need to emphasize an important distinction: elevated expression in tumor tissues does not necessarily correlate with high-risk classification.

2. Risk Group Analysis:

Following your suggestion, we conducted additional analyses comparing gene expression between high-risk and low-risk groups. Our findings reveal:

- SAMM50 and DUSP18 demonstrate significantly higher expression in the low-risk group

- These genes function as protective factors (HR < 1)

- This pattern is consistent with their protective role in tumor progression

3. New Supporting Evidence:

To better illustrate these findings, we have:

- Added a new supplementary figure (S4 Fig) showing the expression patterns across risk groups

- Cross-referenced these results with our previous findings in Fig 2C *(lines 314-319)*

4. Biological Significance:

The inverse correlation between SAMM50/DUSP18 expression and risk classification aligns with their protective nature - higher expression associates with better prognosis (low-risk), while lower expression associates with poorer prognosis (high-risk).

These findings provide a more nuanced understanding of how these genes contribute to endometrial cancer progression and risk stratification.

**Comment 5**

Figure 10N, regarding the impact on in vivo tumor growth of MACC1 downregulation, is not included in the submitted version.

**Response 5**

Thank you for bringing this oversight to our attention. We sincerely apologize for the incomplete figure submission. We have now revised Figure 8 to include:

1. The in vivo tumor growth data demonstrating the impact of MACC1 downregulation

2. Representative images of xenograft tumors

3. Quantitative analysis of tumor volume and weight

4. Statistical comparisons between control and MACC1-knockdown groups

The complete figure now comprehensively illustrates the functional significance of MACC1 in endometrial cancer progression through our in vivo xenograft model experiments.

**Comment 6**

In the discussion, the manuscript would benefit from a correlation between the results observed in the in vitro experiments downregulating MACC1 and what it is known about its function regulating migration, epithelial to mesenchymal transition and cytoskeleton dynamics.

**Response 6**

We sincerely appreciate your valuable suggestion to strengthen the mechanistic discussion of MACC1's role in endometrial cancer. Following your recommendation, we have extensively revised the discussion section to integrate our findings with the established molecular functions of MACC1.

In the revised discussion (Lines 583-597), we have expanded the mechanistic insights as follows:

Our study demonstrates that downregulation of MACC1 significantly curtails the migration and invasion of endometrial cancer cells. This inhibitory effect may be associated with MACC1's well-documented role in facilitating migration and metastasis through modulation of epithelial-mesenchymal transition (EMT) and cytoskeletal dynamics. EMT is characterized by reduced E-cadherin expression and increased mesenchymal markers, and as a pivotal mediator of the transition from epithelial to mesenchymal cell states, the observed decrease in cellular motility following MACC1 knockdown supports its role in regulating EMT. MACC1 can influence the remodeling of the actin cytoskeleton, a regulation crucial for cellular morphology and migration. Our findings reveal that knockdown of MACC1 results in a decline in the invasive capacity of endometrial cancer cells, potentially linked to MACC1's function in cytoskeletal organization. Furthermore, the reduction in tumor cell migration and invasion observed in our experiments aligns with MACC1's established role as a metastasis-promoting factor, providing a mechanistic basis for its prognostic value in endometrial cancer.

**Comment 7**

Even though the authors describe in vivo work in the last part of the results, the methods section of that experiment is described in detailed, and mention Figure 10N which is not included, in the discussion it is written that this study has only been tested experimentally in vitro.

**Response 7**

Thank you for bringing this inconsistency to our attention. We apologize for any confusion caused by the discrepancy between the methods section, results, and discussion. We have made the following corrections:

1. Figure Update:

- Added the complete Figure 8 showing in vivo xenograft data

- Included all relevant animal experimental results

2. Discussion Revision:

- Removed the incorrect statement suggesting "only in vitro testing"

- Added a comprehensive discussion of both in vitro and in vivo findings

3. Results Integration:

- Ensured consistency between methods, results, and discussion sections

- Properly referenced the in vivo experimental data throughout the manuscript

- Maintained alignment with the detailed methods description

This revision ensures that our manuscript accurately represents the complete scope of our research, including both in vitro and in vivo experimental evidence supporting our conclusions about MACC1's role in endometrial cancer.

**Comment 8**

Minor comments:

- Introduction, line 99 – 100, the authors write: “Mitochondria take up substrates from the cytoplasm and use them for important life activities (6).” In this context, it would be better to change “life activities” for “cellular processes”

- Materials and methods, line 186: correct to cell culture.

**Response 8**

We appreciated your constructive comments. We have made detailed revisions to address the above-mentioned errors.

*Line 65-66:*

Mitochondria take up substrates from the cytoplasm and use them for important cellular processes.

*Line 156:*

*Cell culture*
